# The Knowledge, Attitudes, and Practices of Community Pharmacists in their Approach to Antibiotic Use: A Nationwide Survey in Italy

**DOI:** 10.3390/antibiotics8040177

**Published:** 2019-10-07

**Authors:** Francesco Napolitano, Giorgia Della Polla, Caterina De Simone, Chiara Lambiase, Concetta Paola Pelullo, Italo Francesco Angelillo

**Affiliations:** Department of Experimental Medicine, University of Campania “Luigi Vanvitelli”, Via L. Armanni, 5 80138 Naples, Italy; francesco.napolitano2@unicampania.it (F.N.); giorgiadellapolla@gmail.com (G.D.P.); caterinadesimone14@gmail.com (C.D.S.); chiara.lambiase@gmail.com (C.L.); paolapelullo@hotmail.it (C.P.P.)

**Keywords:** antibiotic resistance, antibiotic use, attitudes, community pharmacists, knowledge, Italy, practices

## Abstract

**Background**: This investigation has been carried out to evaluate the knowledge, attitudes, and practices toward antibiotic resistance and antibiotic use among community pharmacists in Italy and to investigate their influencing factors. **Methods**: A cross-sectional telephone survey using a semi-structured interview was performed between September 2018 and April 2019 among a random sample of community pharmacists. **Results**: Almost two thirds (61.5%) correctly answered that the overuse of antibiotics in primary care, hospital settings, and veterinary medicine is a significant cause of antibiotic resistance. Males, those who worked a lower number of hours per week, and those who acquired information through scientific journals and educational activity were more likely to know that the overuse of antibiotics causes antibiotic resistance. More than two thirds of those pharmacists (70.8%) often or always inform the public about the risks of antibiotic resistance. Those who have been practicing for a higher number of years were more likely to act in that way, whereas pharmacy directors and those who did not need additional information on antibiotic resistance were less likely to inform the public. **Conclusions**: Pharmacists need to be aware of the issue of antibiotic resistance and policy makers should promote the implementation of antibiotic use public educational interventions in this setting.

## 1. Introduction

The antibiotics inappropriately used for the treatment of infectious diseases, their overuse in the food production industry, and incorrect patient behaviors, have led to the selection of multi-resistant microorganisms. Indeed, the threat of antibiotic resistance is increasing globally, and this has caused many infectious diseases to become untreatable, with considerable increases in treatment complexity, side effects, disability, and mortality [1,2]. Moreover, it is well established that antibiotic-resistant infectious diseases lead to an increase of healthcare costs for hospitalizations, additional diagnostic investigations, and treatments [3,4,5,6]. The prevention of antibiotic-resistant bacteria’s development and diffusion is a worldwide public health priority and requires a complex approach that includes the participation of healthcare professionals, patients, the agricultural sector, and policy makers. Initiatives and campaigns have been promoted by health organizations across the world to reduce patients’ self-medication of antibiotics and inappropriate prescription in clinical settings [7,8].

Community pharmacists could play an important role in administering and providing recommendations to patients on antibiotic use, since they are a primary source of healthcare information, in the context of limited physician access, and patients often seek their services directly. The Italian Ministry of Health highlighted the importance of the pharmacists’ engagement in order to prevent the misuse of antibiotics through the implementation of public information activities during their activity [9]. As such, their knowledge, attitudes, and behaviors towards antibiotic use can influence their own choices and those of the individuals they encounter. 

Several investigations in different countries have assessed the knowledge, attitudes, and behavior of the general population and physicians regarding antibiotic use [10,11,12,13,14,15]; however, relatively few have been conducted among pharmacists [16,17,18]. To date, there is a general paucity of information in the literature on this topic among Italian community pharmacists. Therefore, this investigation has been carried out to evaluate the level of knowledge, attitudes, and behaviors toward antibiotic resistance and antibiotic use among community pharmacists in Italy and to investigate their influencing factors. 

## 2. Results

Out of 550 community pharmacists who were contacted, 418 agreed to respond with a participation rate of 76%. Table 1 shows the main socio-demographic and professional characteristics of the respondents. The average age was 48.5 years (range 26–79), more than half were female and married, the majority were pharmacy owners, the average number of years in practice was 19.9, and the mean number of hours worked per week was 42.1.

### 2.1. Knowledge

Regarding the level of knowledge, a landslide majority of respondents were aware that the overuse of broad-spectrum antibiotics (98.3%), patient self-medication (96.2%), failure to comply with dosage (93.1%), and non-adherence to the antibiotic course (89.8%) were causes of antibiotic resistance. Almost two thirds (61.5%) correctly answered that the overuse of antibiotics in primary care, hospital settings, and veterinary medicine was a cause of antibiotic resistance. The results of the multivariate logistic regression analysis showed that being male (OR = 0.6; 95% CI 0.39–0.92) and working a lower number of hours per week (OR = 0.97; 95% CI 0.95–0.99) were found to be significantly associated with knowing that the overuse of antibiotics causes antibiotic resistance. Compared with pharmacists who did not receive information or used other sources, the probability of having this knowledge was 4.21 times higher among those who had acquired information through scientific journals (95% CI 1.5–11.82) and was 2.39 times higher among those who had participated in educational activity (95% CI 1.36–3.83) (Model 1 in Table 2).

### 2.2. Attitudes

With regard to attitudes, 75.3% agreed that they should be more engaged in educational interventions on antibiotic use, and almost all (91.7%) believed that they could play an important role in these interventions, with a mean value of 7.8 on a scale of 1 to 10. The results of the multivariate linear regression model built to test the variables associated with this attitude, showed that male pharmacists, those who were younger, those who knew that the overuse of antibiotics in primary care, hospital settings, and veterinary medicine was a cause of antibiotic resistance, and those who worked a higher number of hours for week, were more likely to consider their role in educational interventions on antibiotic use important (Model 2 in Table 2). 

A large proportion of the respondents (89.4%) had a positive attitude regarding the usefulness of information that they provide on antibiotic resistance, with an average value of 7.6 out of 10. The multivariate linear regression analysis showed that male pharmacists, those who have been practicing for a lower number of years, and those who knew that the overuse of antibiotics in primary care, hospital settings, and veterinary medicine was a cause of antibiotic resistance were more likely to have this positive attitude (Model 3 in Table 2).

### 2.3. Practices

Regarding of practices, 41.5% of the sample indicated that they often had received a request for advice on antibiotic treatment by the public, mainly regarding the administration (53.4%), type (50.2%), duration of treatment (43.7%), and side effects (35.7%). In addition, almost all (95%) reported that the public asked for antibiotics without a prescription for the treatment of dental infections (47.7%), cystitis (47.2%), sore throats (41.8%), influenza (42.5%), colds (29%), and fever (23.6%) and the main reasons were lack of time for clinical visits (77.1%), previous treatment experience (45.2%), having antibiotics at home (30.7%), and physician unavailable (24.1%). More than two-thirds (70.8%) often or always inform the public about the importance of following the recommended duration and dosage of antibiotic treatment. Multivariate logistic regression analysis showed that pharmacists who often or always informed the public about the risks of antibiotic resistance (OR = 6.6; 95% CI 3.54–12.31), those who believed the usefulness of information provided on antibiotic resistance (OR = 1.39; 95% CI 1.13–1.72), those who reported that the public sometimes or often asking for advice about antibiotics (OR = 2; 95% CI 1.06–3.74), and those who reported the public’s self-medication (OR = 2.56; 95% CI 1.11–5.91) were more likely to inform the public. Employee pharmacists, compared to owners (OR = 0.38; 95% CI 0.2–0.72), and those who received information by educational activity (OR = 0.31; 95% CI 0.18–0.55), compared to those who did not receive information, were less likely to have this behavior (Model 4 in Table 2).

A total of 15.7% did not inform the public about the risks of antibiotic resistance, and almost half (44.4%) often or always did this. Respondents who had been practicing for a higher number of years (OR = 1.03; 95% CI 1.01–1.06) were more likely to inform the public about the risks of antibiotic resistance. In addition, directors, compared to owners, (OR = 0.5; 95% CI 0.26–0.95), and those who did not need additional information (OR = 0.52; 95% CI 0.3–0.88) were less likely to inform the public (Model 5 in Table 2).

### 2.4. Sources of Information

Respondents were asked to indicate the source of knowledge about antibiotic resistance in their professional practice. Almost all (97.6%) heard about the antibiotic resistance from various sources, mainly from educational activity (61.5%), scientific journals (57%), and the Internet (33.6%). A vast majority (79.3%) felt a need for additional information about antibiotic resistance.

## 3. Discussion

The present research is the first nationwide analysis in Italy to comprehensively assess knowledge, attitudes, and practices surrounding antibiotic use and antibiotic resistance among community pharmacists, and to also assess the effects of several characteristics.

The findings of the survey demonstrated that pharmacists’ knowledge regarding the overuse of antibiotics in primary care, hospital settings, and veterinary medicine as the main cause of antibiotic resistance is lacking. This inadequate knowledge could be translated to a lack of information being disseminated to the public. However, almost all respondents were aware of the main causes of antibiotic resistance, including the overuse of broad-spectrum antibiotics, inconsistent treatment duration, and patient self-medication. The knowledge regarding the main causes of antibiotic resistance was higher than those reported in similar investigations [16,19,20,21]. Considering that health care professionals are envisaged to provide support about antibiotic use, this unsatisfactory level of knowledge highlights the need, in the fight against antibiotic resistance, to implement health promotion programs in order to bridge the gap in knowledge and to revise the existing communicative and educative activities.

Regarding the attitudes, the results showed that a significant proportion of study participants believed that the public’s educational interventions on antibiotic use must be incorporated into their activity and that they could play an important role in those kinds of interventions. This positive perception regarding their prominent role in the delivery of antimicrobial stewardship programs has already been reported in other, similar surveys conducted in different countries [22,23,24]. Therefore, policy makers should plan for greater engagement of pharmacists in educational campaigns for the general population. Indeed, pharmacists are in a privileged position to provide adequate information, as they represent an important link between the physicians and the public, and through their frequent contact with the public, can help to enhance the correct use of antibiotics. Moreover, it should be noted that respondents who more knowledgeable were more likely to express a positive attitude. This reflects the importance of acquiring knowledge for their profession.

In the overall results regarding the practice performed by the pharmacists, more than two thirds of the sample often or always informed the public about the importance of complying with antibiotics’ therapy durations and dosages, and almost half reported that they often or always outlined to them, the risks of antibiotic resistance. This implies that better education is required on antibiotic use and on the risks of antibiotic resistance to improve their practice. That is of particular importance, due the fact that the majority of respondents reported that the public request antibiotics without a prescription and that the most common reasons cited were lack of time for clinical visits, previous treatment experience, and a physician being unavailable. These findings are in line with those reported in a previous survey conducted by some of us in the same geographic area among the general population [22]. The frequency of self-medication was much higher than that observed in Pakistan, where 74.4% of the pharmacy owners have agreed that the public demands antibiotics from them without the prescription [25].

As the present results pointed out, the majority of participants identified educational activity and scientific journals as the two most common sources of information regarding antibiotic resistance, which are considered the most powerful forms of communication of health information. Such sources may provide possible targets for conducting interventions aimed at increasing the knowledge. Indeed, it is very important to acknowledge that these sources have played a vital role in improving the level of knowledge, since when comparing the influence of different sources of information about antibiotic resistance on the level of knowledge, educational activity and scientific journals were significantly associated with pharmacists who knew that the overuse of antibiotics causes antibiotic resistance. This result concurred with the findings of previous studies conducted among different groups of individuals on their level of knowledge related to health issues, which documented the strong impact of acquiring information from these sources [26,27,28,29,30,31]. There is no doubt that this underscored the crucial role of educational activity and scientific journals in improving pharmacists’ knowledge, and therefore, the use of these sources should be encouraged. Furthermore, one-third gained their information from the Internet. This is important because not all information from this source is necessarily correct and reliable, with a greater chance of misleading information and a lack of trustworthy information.

Results from multiple linear and logistic regression analyses showed that several other factors were significant predictors influencing the knowledge, attitudes, and practices of pharmacists. Among the different socio-demographic characteristics of the respondents, age, gender, years of experience, number of hours worked per week, and employment type, were the most important variables. Antibiotic knowledge has been found to be higher in respondents working a lower number of hours worked per week and in males. Those who considered their role in educational interventions on antibiotic use important were more likely to be males and younger, as were respondents who had been practicing for a lower number of years had a positive attitude regarding the usefulness of information that they provided on antibiotic resistance. Those with a higher number of years were more likely to inform the public about the risks of antibiotic resistance. This might be due to that their knowledge in this area relies on experience gained in practice. Finally, directors and employees, compared to owners, respectively, had a more positive attitude regarding the usefulness of information that they provided on antibiotic resistance and were less likely to often or always inform the public about the importance of following the recommended durations and dosages of antibiotic treatments. Participants who reported the public’s self-medication were more likely to often or always inform the public about the importance of complying with therapy duration and dose. This might be because community pharmacists, rather than other health professions, are more likely to perform referrals for health services, combined with the fact that pharmacies are readily accessible to consumers, and therefore, they have the chance to discourage antibiotic self-medication and make the public aware of the risks of antibiotic resistance.

This current survey encountered some limitations that deserve to be taken into account while interpreting the findings. First, the cross-sectional nature of the questionnaire did not enable the researchers to establish the direction of results or cause and effect relationships between the dependent and independent variables. Second, like any other similar surveys using a semi-structured telephone interview, the accuracy of the results was heavily dependent on the honesty of the respondents, since some of them might have provided socially desirable responses, which could impact on participants’ true attitudes and practices. Interviewees were reassured that answers would be kept anonymous for analyses and this could have minimized this bias. Third, the interview’s questionnaire was a new tool and was been previously validated. Because the instrument was not validated, reproducibility and consistency were not evaluated. Despite these limitations, this study provides useful information about the need for more antibiotic education in pharmacy training programs in Italy and the strong points were that it was conducted among a random sample in the whole country with a high response rate, and therefore, the findings may be representative of the entire pharmacist population.

## 4. Materials and Methods

### 4.1. Setting and Sampling

A cross-sectional survey among a nationally representative sample of community pharmacists in Italy was performed between September 2018 and April 2019. The sample was drawn with a two-stage random cluster sampling strategy. In the first stage 500 community pharmacies located in the three geographical areas (northern, central, and southern and islands) were randomly selected from the national list. In the second stage, from each pharmacy, only one pharmacist (owner, employee, or director) who directly dealt with the public was recruited for interview. The minimum calculated sample size was 323, which was determined using a 70% expected knowledge rate regarding the causes of antibiotic resistance, a confidence interval of 95%, and an error rate of 5%. Considering a non-response rate of 20%, a target sample of 405 pharmacists was finally determined.

### 4.2. Procedure

Data were collected by a semi-structured telephone interview performed by members of the research team that had been trained in the purpose of the study and data collection techniques, and who were familiar with the contents. Prior answering the questions, the nature and the purpose of the study was explained to the pharmacists, as were the data collection methods, that participation was voluntary, and that they could stop at any stage without penalty if they did not wish to continue. Participants were told that the data were collected, processed, and analyzed anonymously, and they were assured of the anonymity and confidentiality of their responses. The pharmacists provided verbal consent after learning about the study and its purpose. No remuneration or gifts were given to participants for taking part in the survey.

### 4.3. Survey Instrument

A two-page questionnaire was used to collect the data, composed of five sections. The first section collected socio-demographic and professional characteristics, including gender, age, marital status, year of graduation, number of years in practice, and practice size. The second section assessed the pharmacists’ knowledge about the causes of antibiotic resistance, such as overuse of antibiotics, self-medication, and non-compliance with recommended dose or duration of treatment. The knowledge about the overuse of antibiotics as the cause of antibiotic resistance was explored by presenting the following three propositions: (1) The overuse of antibiotics in primary care is a cause of antibiotic resistance; (2) the overuse of antibiotics in hospital settings is a cause of antibiotic resistance; (3) the overuse of antibiotics in veterinary medicine is a cause of antibiotic resistance. These questions had “Yes,” “No,” and “Do not know” response options. The third section included three questions measuring the attitudes towards their role in public educational interventions on antibiotic use. Two questions were scored using a numerical 10-point Likert-type scale with higher values corresponding to a stronger attitude, and one used a 5-point Likert-type scale with response options ranging from “Strongly disagree” to “Strongly agree.” The fourth section collected information about antibiotic dispensation practices and communication with the public regarding antibiotic use and antibiotic resistance. The questions were measured either using a 5-point Likert-type scale ranging from “Never” to “Always” or through multiple-choice alternatives. The fifth section queried about their sources of information about the antibiotic use and if they would prefer to learn more.

The survey questions were pretested to a random sample of 20 pharmacists that were not part of the study to ensure the clarity, validity, and ease of use. Ethical approval for the study protocol and questionnaire was sought from the Ethics Committee of the Teaching Hospital of the University of Campania “Luigi Vanvitelli” (approval number 767).

### 4.4. Statistical Analysis

Data analyses were conducted using Stata statistical software version 15 [32]. Primarily, descriptive statistics were used to summarize all information. Secondly, univariate analysis was performed, using chi-square and Student’s *t*-tests, to evaluate the association between the outcomes of interest and the independents variables. The variables with *p*-values ≤ 0.25 at univariate analysis were included into multivariate linear and logistic regression models. Finally, a multivariate stepwise procedure was used to estimate the independent association between potential predictors and the following outcomes of interest: pharmacists who known that the overuse of antibiotics in primary care, hospital settings, and veterinary medicine was a cause of antibiotic resistance (Model 1); pharmacists who believed that they could play an important role in educational interventions on antibiotic use (Model 2); pharmacists who believed that it is important that they inform the public about the antibiotic resistance (Model 3); pharmacists who believed that it is important to inform the public about the duration and dosage recommended for the antibiotic treatment (Model 4); and pharmacists who informed often or always the public about the risks of antibiotic resistance (Model 5). The following independent variables were included in all Models: age (continuous), gender (male = 0; female = 1), number of years since degree (continuous), number of years in practice (continuous), number of hours worked per week (continuous), employment type (owner = 1; employee = 2; director = 3), sources of information (none = 1; scientific journals = 2; educational activity = 3; other = 4), and need of additional information about antibiotic resistance (no = 0; yes = 1). Moreover, the following variables were also included: knowledge that the overuse of antibiotics in primary care, hospital settings, and veterinary medicine is a significant cause of antibiotic resistance (no = 0; yes = 1) in Models 2 to 5; pharmacists who believed that it is important that they inform the public about the antibiotic resistance (continuous), pharmacists who believed that they could play an important role in educational interventions on antibiotic use (continuous), pharmacists who indicated that the public often ask for advice on antibiotic treatment (no = 0; yes = 1), and pharmacists who indicated that the public reported self-medication (no = 0; yes = 1) in Models 4 and 5; pharmacists who often or always informed the public about the risks of antibiotic resistance (no = 0; yes = 1) in Model 4; and pharmacists who often or always informed the public about the importance of following the recommended duration and dosage of antibiotic treatment in Model 5. The significance level was set at 0.2 for entering and at 0.4 for removing the variables in the stepwise logistic and linear regression models. Odds ratios (ORs) and their 95% confidence intervals (CIs) were estimated from the logistic regression models. Standardized regression coefficients (β) were presented for the linear regression models. All statistical tests were two-tailed, and results were considered to be statistically significant if the *p*-values were less than or equal to 0.05.

## 5. Conclusions

This nationwide study reveals the current situation about the knowledge, attitudes, and practices of the Italian community pharmacists in their approach to antibiotic use. It is imperative that pharmacists become aware of the issue of antibiotic resistance and policy makers should promote the implementation of public educational interventions in this setting.

## Figures and Tables

**Table 1:** Socio-demographic and professional characteristics of the study population.

	N	%
Age, years	48.5 ± 10.5 (26–79) *	
Gender		
Male	186	44.5
Female	232	55.5
Marital status		
Married	236	56.6
Other	181	43.4
Number of years since degree	22.1 ± 10.7 (1–54) *	
Number of years in practice	19.9 ± 10.9 (1–45) *	
Number of hours worked per week	42.1 ± 9 (1–72) *	
Role in the pharmacy		
Owner	241	57.4
Employee	113	26.9
Director	66	15.7

* Mean ± standard deviation (range).

**Table 2:** Multivariate logistic and linear regression analysis to characterize factors associated with the different outcomes.

**Variable**	**OR**	**SE**	**95% CI**	***p***
Model 1. Knowledge that the overuse of antibiotics in primary care, hospital settings, and veterinary medicine is a significant cause of antibiotic resistance				
Log likelihood = −249.33, χ^2^ = 34.51 (5 df), *p* < 0.0001				
Source of information				
None	1 *			
Scientific journals	4.21	0.15	1.5–11.82	0.02
Educational activity	2.39	0.6	1.36–3.83	0.002
Male	0.6	0.13	0.39–0.92	0.021
Pharmacists who worked a lower number of hours per week	0.97	0.01	0.95–0.99	0.038
Pharmacists who needed additional information	0.61	0.17	0.35–1.04	0.07
**Variable**	**COEFF.**	**SE**	**t**	***p***
Model 2. Pharmacists who believed that they could play an important role in educational interventions on antibiotic use				
F = 47.04, R^2^ = 0.46%, adjusted R^2^ = 0.45%, *p* < 0.0001				
Pharmacists who believed that it is important that they inform the public about the antibiotic resistance	0.54	0.03	15.77	<0.001
Pharmacists who worked a higher number of hours per week	0.02	0.006	2.82	0.005
Younger pharmacists	−0.01	0.005	−2.41	0.017
Knowledge that the overuse of antibiotics in primary care, hospital settings, and veterinary medicine is a significant cause of antibiotic resistance	0.22	0.11	1.88	0.061
Employment type				
Owner	1 *			
Director	−0.29	0.15	−1.87	0.063
Employee	−0.14	0.14	−0.99	0.325
Male	0.21	0.11	1.85	0.064
**Variable**	**COEFF.**	**SE**	**t**	***p***
Model 3. Pharmacists who believed that it is important that they inform the public about the antibiotic resistance				
F = 59.47, R^2^ = 0.47%, adjusted R^2^ = 0.46%, *p* < 0.0001				
Pharmacists who believed that they could play an important role in educational interventions on antibiotic use	0.74	0.04	17.11	<0.001
Pharmacists who did not need additional information	−0.37	0.15	−2.45	0.015
Knowledge that the overuse of antibiotics in primary care, hospital settings, and veterinary medicine is a significant cause of antibiotic resistance	0.27	0.13	2.15	0.032
Employment type				
Owner	1 *			
Employee	0.27	0.13	1.82	0.069
Lower number of years since degree	−0.01	0.01	−1.04	0.298
Older pharmacists	0.01	0.01	0.94	0.345
**Variable**	**OR**	**SE**	**95% CI**	***p***
Model 4. Pharmacists who often or always informed the public about the importance of following the recommended duration and dosage of antibiotic treatment				
Log likelihood = −177.81, χ^2^ = 146.7 (11 df), *p* < 0.0001				
Pharmacists who often or always informed the public about the risks of antibiotic resistance	6.6	2.1	3.54–12.31	<0.001
Source of information				
None	1 *			
Educational activity	0.31	0.09	0.18–0.55	<0.001
Pharmacists who believed that it is important that they inform the public about the antibiotic resistance	1.39	0.15	1.13–1.72	0.002
Employment type				
Owner	1 *			
Employee	0.38	0.12	0.2–0.72	0.003
Director	0.63	0.23	0.31–1.28	0.2
Pharmacists who indicated that the public reported self-medication	2.56	1.09	1.11–5.91	0.028
Pharmacists who indicated that the public often ask for advice on antibiotic treatment	2	0.64	1.06–3.74	0.031
Pharmacists who have been practicing for a lower number of years	0.98	0.01	0.95–1.01	0.056
Pharmacists who needed additional information	0.49	0.19	0.22–1.05	0.066
Pharmacists who believed that they could play an important role in educational interventions on antibiotic use	1.15	0.14	0.9–1.47	0.266
Knowledge that the overuse of antibiotics in primary care, hospital settings, and veterinary medicine is a significant cause of antibiotic resistance	0.76	0.22	0.44–1.33	0.341
**Variable**	**OR**	**SE**	**95% CI**	***p***
Model 5. Pharmacists who often or always informed the public about the risks of antibiotic resistance				
Log likelihood = −248.36, χ^2^ = 55.37 (11 df), *p* < 0.0001				
Pharmacists who have been practicing for a higher number of years	1.03	0.01	1.01–1.06	0.005
Pharmacists who did not need additional information	0.52	0.14	0.3–0.88	0.015
Employment type				
Owner	1 *			
Director	0.5	0.16	0.26–0.95	0.035
Employee	0.72	0.2	0.42–1.25	0.248
Pharmacists who believed that it is important that they inform the public about the antibiotic resistance	1.2	0.11	1.01–1.43	0.052
Source of information				
None	1 *			
Scientific journals	0.53	0.19	0.26–1.06	0.071
Educational activity	0.81	0.2	0.5–1.31	0.394
Pharmacists who indicated that the public often ask for advice on antibiotic treatment	1.39	0.3	0.91–2.14	0.126
Knowledge that the overuse of antibiotics in primary care, hospital settings, and veterinary medicine is a significant cause of antibiotic resistance	1.4	0.33	0.88–2.21	0.153
Pharmacists who believed that they could play an important role in educational interventions on antibiotic use	1.15	0.12	0.94–1.41	0.169
Pharmacists who worked a higher number of hours per week	1.02	0.01	0.99–1.04	0.207

* Reference category.
